# Supplementary material for: Understanding WT1 Alterations and Expression Profiles in Hematological Malignancies
Source: Cancers (Basel). 2023 Jul 4;15(13):3491. doi: 10.3390/cancers15133491 (PMC10340278; doi:10.3390/cancers15133491)
Supplement: Supplementary file 1 [file cancers-15-03491-s001.zip › cancers-2411712-supplementary.pdf]

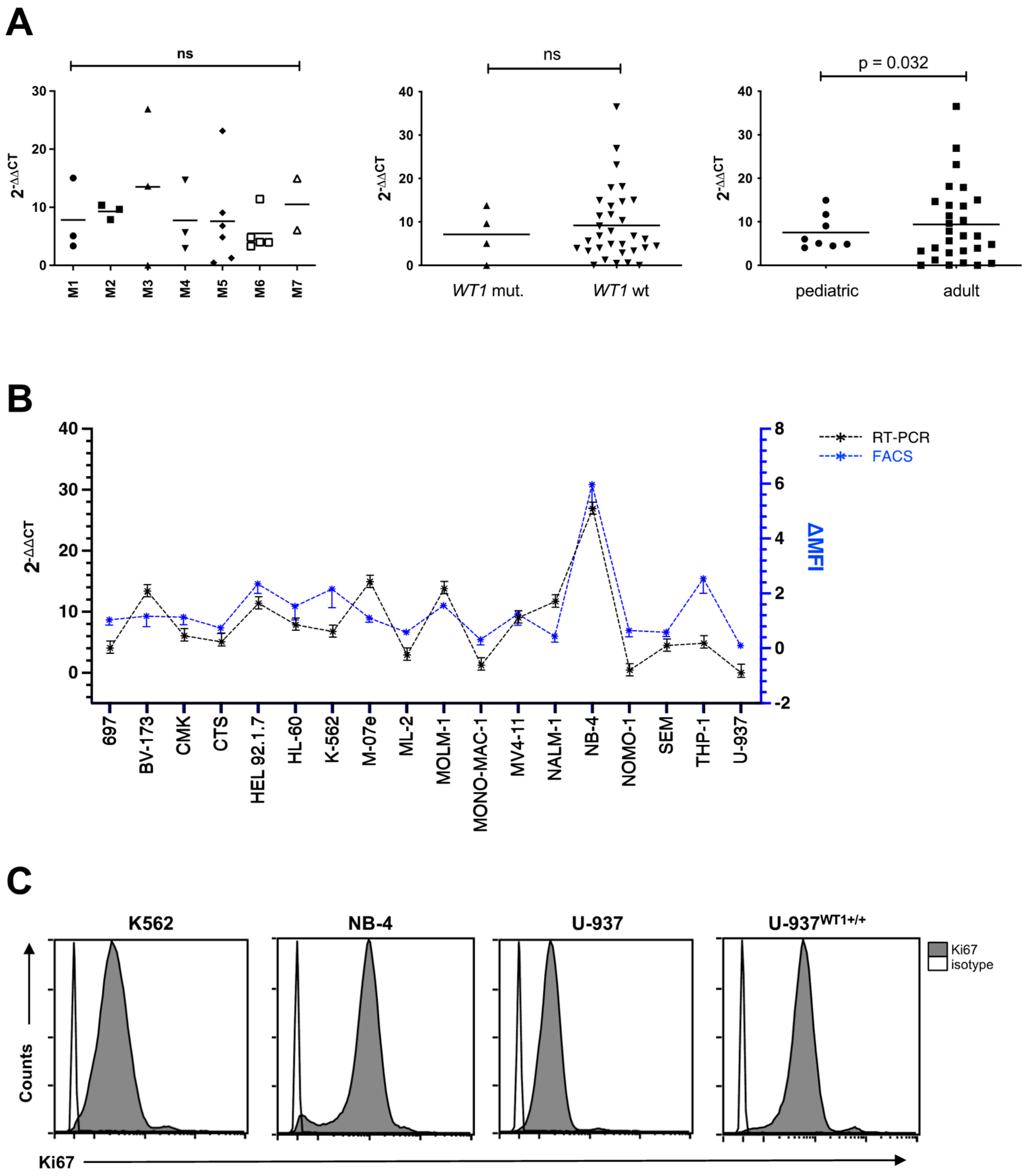

Figure S1. Association of *WT1* mRNA expression with different leukemic features and its correlation with WT1 protein. (A) Total mRNA was extracted from different cell lines and reverse-transcribed into cDNA. The cDNA was used as template for RT-PCR in combination with selective primer pairs for *WT1* exon 2 and *ABL1*. *WT1*'s cycle threshold was normalized to ABL1 (ΔCt) and HEK293 cells (ΔΔCt). The fold change expression normalized to that of detected in HEK293 cells was reported (2^-ΔΔCt). The graph shows mean values ± SD of three biological replicates. **(B)** Expression of *WT1* mRNA measured in panel A was compared to the expression of WT1 protein measured as follows. One day after splitting cultures, $1x10^6$ cells per line were fixed and permeabilized using paraformaldehyde and methanol as described in the methods. The intracellular staining was performed for *WT1* antibody and its isotype control as well as Ki67 as a technical positive control (data for Ki67 not shown). Population's MFI was normalized to that of expressed in isotype control via subtraction ($\Delta MFI = MFI_{WT1} - MFI_{isotype}$). The graph shows mean ± SE of two to three biological replicates. **(C)** Histograms of four examples of cell lines delineating the percentage of the Ki67 intracellular protein. Here, the Ki67 staining was performed as a technical positive control for WT1 flow cytometry as described in Figure 1. One day after splitting cultures, $1x10^6$ cells per line were fixed and permeabilized using paraformaldehyde and methanol as described in the methods. The intracellular staining was performed for Ki67 antibody and its isotype control. Abbreviations: *WT1, Wilms Tumor 1*; cDNA, complementary DNA; RT-PCR, real-time PCR; *ABL*, *Abelson tyrosine-protein kinase*; MFI, mean florescence intensity.

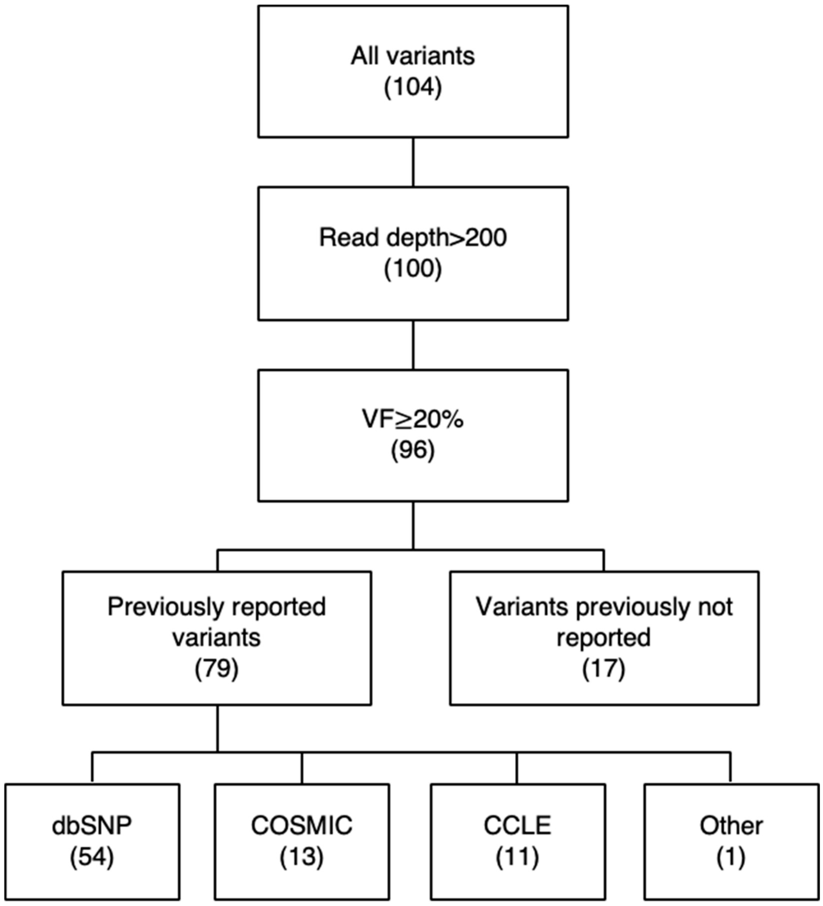

Figure S2. Validation pipeline for variants detected via NGS.
Abbreviations. VF, variant frequency; NGS, next generation sequencing. ClinVar (dbSNP) database accessed at https://www.ncbi.nlm.nih.gov/clinvar; COSMIC database accessed at https://cancer.sanger.ac.uk/cosmic CCLE database accessed at https://depmap.org.

Table S1. Overview of cell lines included in this data set and performed analyses for each.

#	Cell line	NGS	RT-PCR	Sanger	FC	FA	Disease	Subtype	Fusion transcripts[1]	Cell Origin	Source
1	697	-	+	-	+	+	ALL	B-ALL	t(1;19)*TCF3::PBX1*	Pediatric	Cultivated cells[2]
2	AML-193	+	-	-	-	-	AML	M5	t(1;12)*RAP1B::RAP1A*	Pediatric	Cell pellet[3]
3	AP-1060	-	+	-	-	-	AML	M3	t(15;17)*PML::RARA*, t(12;15)*ETV6::NTRK3*	Adult	Cell pellet
4	BJAB	-	-	-	+	-	Lymphoma	NHL (Burkitt)	t(11;17)*KMT2A::CLTC*	Pediatric	Cultivated cells
5	BV-173	-	+	-	+	+	CML	CML in BC	t(9;22)*BCR::ABL1*	Adult	Cultivated cells
6	CMK	-	+	+	+	+	AML	M7	t(18;17)*ADNP2::NSF*, t(12;1)*RAP1B::RAP1A*	Pediatric	Cultivated cells
7	CTS	+	+	+	+	+	AML	M1	t(6;11)*KMT2A::AFDN*	Pediatric	Cultivated cells
8	ELF-153	+	-	-	-	-	AML	M7	No record of fusion transcripts	Adult	Cell pellet
9	F-36P	-	+	-	-	-	AML	M6	*t(19;21)ZNF625-ZNF20::MIR99AHG, t(19;21)ZNF625::MIR99AHG, Inv(16)ANKS3::SEPT12, t(21;19)MIR99AHG::PTBP1, Inv(19)EPS15L1::PALM3, Inv(9)DOCK8::RFX3-AS1, t(21;19)MIR99AHG::PTBP1*	Adult	Cell pellet
10	FKH-1	+	+	+	-	-	AML	M4	t(9;12)*ETV6::ABL1*, t(6;9)*DEK::NUP214*	Adult	Cell pellet
11	GF-D8	+	-	-	-	-	AML	M1	No record of fusion transcripts	Adult	Cell pellet
12	Granta-519	-	-	-	+	-	Lymphoma	B-NHL	t(8;11)*RPS17P14::RPS17*	Adult	Cultivated cells
13	HEL 92.1.7	+	+	-	+	+	AML	M6	Inv(19)CDC37::IL27RA, t(20;19)TRPC4AP::ACSBG2, Inv(1)MTF2::DR1, Inv(9)FREM1::MPDZ, t(20;19)ITCH::CATSPERD, Inv(20)TTI1::TRPC4AP, t(20;19)RALGAPB::AP1M2, Inv(2)GTDC1::ZEB2	Young adult	Cultivated cells
14	HL-60	+	+	+	+	+	AML	M2	t(5;16)*CYFIP2::PLCG2*	Young adult	Cultivated cells
15	HNT-34	-	+	-	-	-	AML	M4	t(9;22)*BCR::ABL1*	Adult	Cell pellet
16	HT-93	+	+	+	-	-	AML	M3	t(15;17)*PML::RARA*, t(1;12)*ETV6-ABL2*	Adult	Cell pellet
17	K-562	+	+	+	+	+	CML	CML in BC	t(9;22)*BCR-ABL*	Adult	Cultivated cells
18	KASUMI-6	+	+	+	-	+	AML	M2	Inv(13)*UPF3A::CDC16*	Adult	Cultivated cells
19	KG-1	+	+	+	-	-	AML	M6	t(12;8)*FGFR1OP2::FGFR1*	Adult	Cell pellet
20	KG-1a	+	-	-	-	-	AML	M6	t(12;8)*FGFR1OP2::FGFR1*	Adult	Cell pellet
21	KMOE-2	+	-	-	-	-	AML	M6	No record of fusion transcripts	Pediatric	Cell pellet
22	KU-812	-	-	-	+	+	CML	CML in BC	t(9;22)*BCR-ABL*	Adult	Cultivated cells
23	LAMA-84	-	-	-	+	+	CML	CML in BC	t(9;22)*BCR-ABL*	Young adult	Cultivated cells
24	M-07e	+	+	+	+	+	AML	M7	Inv(16)CBFA2T3::GLIS2, t(2;19)ANO7-DHDH, t(9;2)*MELK::RAPGEF4*	Pediatric	Cultivated cells
25	ME-1	+	-	+	+	-	AML	M4Eo	Inv(16)*CBFB::MYH11*	Adult	Cultivated cells
26	Mino	-	-	-	+	-	Lymphoma	MCL	t(11;14) *IGH::CCND1*	Adult	Cultivated cells
27	MKPL-1	+	-	-	-	-	Lymphoma	MCL	t(3;5)*RBM6::CSF1R*,t(11;14)*IGH::CCND1*	Adult	Cell pellet
28	ML-2	-	+	+	+	+	AML	M4	t(6;11)*KMT2A::AFDN*	Young adult	Cultivated cells
29	MOLM-1	+	+	+	+	+	CML	CML in BC	t(9;22)*BCR-ABL*	Adult	Cultivated cells

30	MOLM-13	-	+	-	-	-	AML	M5a	t(9;11)*KMT2A::MLLT3*	Young adult	Cell pellet
31	MOLM-14	+	-	-	+	+	AML	M5	t(9;11)*KMT2A::MLLT3*	Young adult	Cultivated cells
32	MOLM-20	+	-	-	-	-	CNL	-	t(4;11)*KMT2A::SEPT11*	Adult	Cell pellet
33	MONO-MAC-1	+	+	-	+	+	AML	M5	t(9;11)*KMT2A::MLLT3*	Adult	Cultivated cells
34	MONO-MAC-6	-	-	-	+	+	AML	M5	t(9;11)*KMT2A::MLLT3*	Adult	Cultivated cells
35	MUTZ-2	+	-	-	-	-	AML	M2	No record of fusion transcripts	Adult	Cell pellet
36	MUTZ-3	+	-	-	-	-	AML	M4	Inv(17)*BPTF::LRRC37A2,*<br>*Inv(19)*RPL36::RPL36P16	Young adult	Cell pellet
37	MV4-11	+	+	-	+	+	AML	M5	t(9;11)*KMT2A::AFF1*	Pediatric	Cultivated cells
38	NALM-1	-	+	-	+	+	CML	CML in BC	t(9;22)*BCR-ABL*	Pediatric	Cultivated cells
39	NB-4	+	+	+	+	+	AML	M3	t(15;17)*PML::RARA*	Young adult	Cultivated cells
40	NOMO-1	+	+	+	+	+	AML	M5a	t(9;11)*KMT2A::MLLT3*	Young adult	Cultivated cells
41	OCI-AML1	+	-	-	-	-	AML	M4	No record of fusion transcripts	Adult	Cell pellet
42	OCI-AML2	-	-	-	+	+	AML	M4	t(6;11)*KMT2A::AFDN*	Adult	Cultivated cells
43	OCI-AML3	+	-	-	+	+	AML	M4	t(12:6)*GAPDH::RPS18*, t(8;6)NDRG1::RPS18,	Adult	Cultivated cells
44	OCI-AML4	-	+	-	-	-	AML	M4	Inv(19)LSM14A::ZNF416, t(11;19)*KMT2A::MLLT1*	Young adult	Cell pellet
45	OCI-AML5	+	-	-	-	-	AML	M4	t(5;6)*RPL37::RPL37P15*	Adult	Cell pellet
46	OCI-M1	+	-	-	-	-	AML	M6	t(11;17)*DISC1FP1::STX8*, Inv(6)*KIF13A::TPMT*,<br>t(8;17)*ANK1::CCDC144NL*,<br>t(16;6)*ANKRD11::THBS2*, Inv(19)*PTPRS::ZNRF4*	Adult	Cell pellet
47	OCI-M2	-	+	-	-	-	AML	M6	t(21;21)*RUNX1::TSPEAR*	Adult	Cell pellet
48	REH	-	-	-	+	+	ALL	B-ALL	t(12;21)*ETV6::RUNX1*	Pediatric	Cultivated cells
49	RS4-11	-	+	-	-	-	ALL	B-ALL	t(9;11)*KMT2A::AFF1*	Young adult	Cell pellet
50	SEM	-	+	-	+	+	ALL	B-ALL	t(9;11)*KMT2A::AFF1*	Pediatric	Cultivated cells
51	SET-2	-	-	-	+	+	AML	M7	t(9;9)*ADGRE5::NOL6*, t(11;7)*FTH1::ACTB*	Adult	Cultivated cells
52	SKM-1	-	+	-	-	-	AML	M5	Inv(19)*DNMT1::TYK2,* t(19;13)*SSBP4::NUP58*,<br>t(9;15)*RPS6::B2M*	Adult	Cell pellet
53	SKNO-1	+	+	+	-	-	AML	M2	t(8;21)*RUNX1::RUNX1T1* (AML1-ETO)	Young adult	Cell pellet
54	TF-1	+	+	+	-	-	AML	M6	t(16;20)*CBFA2T3::ABHD12*	Young adult	Cell pellet
55	THP-1	+	+	+	+	+	AML	M5	t(9;11)*KMT2A::MLLT3*	Pediatric	Cultivated cells
56	U-937	+	+	+	+	+	Lymphoma	IBL	t(10;11)*MLLT10::PICALM*	Adult	Cultivated cells
57	UOC-M1	+	+	+	-	-	AML	M1	No record of fusion transcripts	Adult	Cell pellet
58	UT-7	-	-	-	-	-	AML	M7	No record of fusion transcripts	Adult	Cell pellet
59	YNH-1	-	+	+	-	-	AML	M1	t(16;21)*FUS::ERG*	Adult	Cell pellet

Abbreviations. NGS, next generation sequencing; FC, flow cytometry; FA, fragment-length analysis; mat., material; ALL, acute lymphoblastic leukemia; AML acute myeloblastic leukemia; NHL, non-Hodgkin's lymphoma; CML in B, chronic myeloblastic leukemia in blast crisis; CNL, chronic neutrophilic leukemia; MCL, mantel cell lymphoma; IBL, immunoblastic lymphoma or histiocytic lymphoma; AMKL, acute megakaryocytic leukemia.

[1]Expression of fusion transcripts detected by RT-PCR (DSMZ, The LL-100 Panel, PMID: 31160637) or RNA sequencing (CCLE, DepMap portal). For the RNAseq data, the junction reads of 10 and above are included in this table. For fusions with lower read counts, please refer to CCLE database at: https://depmap.org/portal/. [2]Before extracting genomic material from cell cultures all cell lines were validated using STR analysis; [3]Cell pellets were received from DSMZ-German collection of microorganisms and cell culture GmbH (Braunschweig, Germany).

Table S2. *WT1* and *ABL1* primer pairs and probes used for RT-PCR.

Target	Location	Direction	Primer sequence
WT1	Exon 2	forward	5'-GTTACAGCACGGTCACCTTC-3'
		reverse	5'-CCATGGGATCCTCATGCTTG-3'
		probe	5'-FAM-GCAGTTCCCCAACCACTCATT-BHQ1-3'
ABL1	Exon 1	forward	5'- GGGTCCACACTGCAATGTTT-3'
	Exon 2	reverse	5'-CCAACGAGCGGCTTCAC-3'
		probe	5'-FAM-TCAGATGCTACTGGCCGCTGAAGG-BHQ1-3'

Table S3. Overview of gene sequenced with TruSight™ myeloid sequencing panel.

#	Gene	Status in current data set[1]		#	Gene	Status in current data set
1	*ABL1*	Wild type		28	*JAK3*	Wild type
2	*ASXL1*	Altered		29	*KDM6A*	Wild type
3	*ATRX*	Altered		30	*KIT*	Altered
4	*BCOR*	Altered		31	*KMT2A*	Wild type
5	*BCORL1*	Altered		32	*KRAS*	Altered
6	*BRAF*	Wild type		33	*MPL*	Wild type
7	*CALR*	Wild type		34	*MYD88*	Wild type
8	*CBL*	Wild type		35	*NOTCH1*	Altered
9	*CBLB*	Wild type		36	*NPM1*	Altered
10	*CBLC*	Wild type		37	*NRAS*	Altered
11	*CDKN2A*	Wild type		38	*PDGFRA*	Wild type
12	*CEBPa*	Altered		39	*PHF6*	Altered
13	*CSF3R*	Altered		40	*PTEN*	Altered
14	*CUX1*	Wild type		41	*PTPN11*	Altered
15	*DNMT3A*	Altered		42	*RAD21*	Wild type
16	*ETV6/TEL*	Altered		43	*RUNX1*	Altered
17	*EZH2*	Altered		44	*SETBP1*	Altered
18	*FBXW7*	Wild type		45	*SF3B1*	Altered
19	*FLT3*	Altered		46	*SMC1A*	Wild type
20	*Gata1*	Wild type		47	*SMC3*	Wild type
21	*GATA2*	Altered		48	*SRSF2*	Wild type
22	*GNAS*	Wild type		49	*STAG2*	Altered
23	*HRAS*	Wild type		50	*TET2*	Altered
24	*IDH1*	Wild type		51	*TP53*	Altered
25	*IDH2*	Wild type		52	*U2AF1*	Altered
26	*IKZF1*	Wild type		53	*WT1*	Altered
27	*JAK2*	Altered		54	*ZRSR2*	Wild type

[1]For detailed description on detected alterations in each gene, refer to Table S4.

Table S4. *WT1* primer pairs and amplification strategies used for Sanger Sequencing.

Location	#	Direction	Primer sequence	Polymerase	Amplification Method
5'UTR and its upstream region	**1**	forward	5‘- ACTAAGTGCTGCTGACTCCA -3‘	Q5® Hot Start High-Fidelity	Touch-down amplification
		reverse	5‘- TCAGGTAAGCAGTGAGTCCG -3‘		
	**2**	forward	5‘- GACTGAGTTCTTTCTGCGCT -3‘	Q5® Hot Start High-Fidelity	Touch-down amplification
		reverse	5‘- TGAACTCCTTACCCCAGCTG -3‘		
	**3**	forward	5‘- TTGGGTTGAAGAGGAGGGTG -3‘	Q5® Hot Start High-Fidelity	Touch-down amplification
		reverse	5‘- GGACTCCCTGCTGCTCTG -3‘		
Exon 1	**4**	forward	5‘- GGACTTCCTCTTGCTGCAGG -3‘	Q5® Hot Start High-Fidelity	Touch-down amplification
		reverse	5‘- TTGATGAAGGAGTGAGGCGG -3‘		
	**5**	forward	5‘- GACGTGCGGGACCTGAAC -3‘	Q5® Hot Start High-Fidelity	Touch-down amplification
		reverse	5‘- TTGGGAAGCAGCTGGGTAAG -3‘		
Exon 2	**6**	forward	5‘- TCTTCCTGCCGAAAGTCCTG -3‘	ALLin™ Hot Start Taq	Standard amplification
		reverse	5‘- AACGTACTTGGGATGGAGGG -3‘		
Exon 3	**7**	forward	5‘- GGGGAATGCAAAGTGAGAGG -3‘	ALLin™ Hot Start Taq	Standard amplification
		reverse	5‘- TAGTAGAGTGGAGTCGAGGC -3‘		
Exon 4	**8**	forward	5‘- TGGAAAATGTGGAGGCTTGC -3‘	ALLin™ Hot Start Taq	Standard amplification
		reverse	5‘- ATTTGGATCCCGGACTTTGC -3‘		
Exon 5	**9**	forward	5‘- TTGAGGGGCTTTTCACTGGA -3‘	ALLin™ Hot Start Taq	Standard amplification
		reverse	5‘- CCATTTTGTCCCCAGCATGT -3‘		
Exon 6	**10**	forward	5‘- TCTAAAGTGGCCCCATGGAG -3‘	ALLin™ Hot Start Taq	Standard amplification
		reverse	5‘- AAAGGAGCCTGCAGTGAAGA -3‘		
Exon 7	**11**	forward	5‘- GGAGTGTGAATGGGAGTGGT -3‘	ALLin™ Hot Start Taq	Standard amplification
		reverse	5‘- CAGAATGCAAAATGGCCCCA -3‘		
Exon 8	**12**	forward	5‘- CCCTGAGCTCCCATTCATTTG -3‘	ALLin™ Hot Start Taq	Standard amplification
		reverse	5‘- TGTGGTTTGCAGGGGAAATG -3‘		
Exon 9	**13**	forward	5‘- TCAGTGATTCTTGTTGGGCC -3‘	ALLin™ Hot Start Taq	Standard amplification
		reverse	5‘- GCACACTCCAGCCATTCTTT -3‘		
Exon 10	**14**	forward	5‘- TGGTTAGCTCAGGGACAGAA -3‘	ALLin™ Hot Start Taq	Standard amplification
		reverse	5‘- TAGTTTCCAGAAGCACCGGT -3‘		

Table S5. Overview of genomic alterations detected *via* NGS.

Gene*	Variant (DNA)	Variant (AA)	VF (%)	Read	Cell Line	dbSNP/COSMIC	Relevance	Reference
TP53	c.817C>T	p.Arg273Cys	99.55	224	CTS	rs121913343	Pathogenic	ClinVar[1]
	c.31G>C	p.Glu11Gln	57.36	652	FKH-1	rs201382018	VUS	ClinVar
	c.711G>A	p.Met237Ile	100,00	968	GF-D8	rs587782664	Pathogenic	ClinVar
	c.700T>G	p.Tyr234Asp	99,79	2874	KASUMI-6	rs864622237	Likely pathogenic	ClinVar
	c.814G>A	p.Val272Met	99.79	481	KMOE-2	rs121912657	Pathogenic	ClinVar
	c.905_919+32del47	intronic variant	96.42	642	ME-1	ACH-000439	VUS	CCLE
	c.637C>T	p.Arg213Ter	54.13	351	MKPL-1	rs397516436	Pathogenic	ClinVar
	c.517G>A	p.Val173Met	49.02	2473	MOLM-16	rs876660754	Pathogenic	ClinVar
	c.713G>C	p.Cys238Ser	48.73	2473	MOLM-16	rs730882005	Likely pathogenic	ClinVar
	c.742C>T	p.Arg248Trp	99.38	5182	MV4-11	rs121912651	Pathogenic	ClinVar
	c.743G>A	p.Arg248Gln	99.11	1353	NB-4	rs11540652	Pathogenic	ClinVar
	c.723delC	p.Cys242AlafsTer5	98.55	3368	NOMO-1	rs730882019	Pathogenic	ClinVar
	c.434T>G	p.Leu145Arg	47.21	754	OCI-M1	rs587782197	Likely pathogenic	ClinVar
	c.743G>A	p.Arg248Gln	99.1	2449	SKNO-1	rs11540652	Pathogenic	ClinVar
	c.520_545del26	p.Arg174LeufsTer3	90.5	968	THP-1	COSM13483	Pathogenic	COSMIC[2]
	c.559+1G>A	intronic variant	99.12	340	U-937	rs1131691042	Likely pathogenic	ClinVar
ASXL1	c.1773C>A	p.Tyr591Ter	32.81	1262	K-562	rs371369583	Pathogenic	ClinVar
	c.3692C>T	p.Ser1231Phe	67.9	5560	ME-1	rs74638057	Benign	ClinVar
	c.1954G>A	p.Gly652Ser	55.08	325	MKPL-1	rs3746609	Benign	ClinVar
	c.1954G>A	p.Gly652Ser	57.55	351	MOLM-16	rs3746609	Benign	ClinVar
	c.1773C>A	p.Tyr591Ter	54.06	1613	MOLM-20	rs371369583	Pathogenic	ClinVar
	c.3692C>T	p.Ser1231Phe	49.33	3430	MOLM-20	rs74638057	Benign	ClinVar
	c.4178_4179delTG	p.Leu1393ArgfsTer30	40.71	280	MONO-MAC-1	ACH-001129	Pathogenic	CCLE
	c.3306G>T	p.Glu1102Asp	31.76	2371	MUTZ-3	rs139115934	Benign	ClinVar
	c.2077C>T	p.Arg693Ter	49.11	902	NOMO-1	rs373221034	Pathogenic	ClinVar
	c.1773C>G	p.Tyr591Ter	52.5	2162	OCI-AML5	rs371369583	Pathogenic	ClinVar
	c.3306G>T	p.Glu1102Asp	35.21	1153	OCI-M1	rs139115934	Benign	ClinVar
NRAS	c.38G>T	p.Gly13Val	48.46	1950	AML-193	rs121434596	Likely pathogenic	ClinVar
	c.38G>A	p.Gly13Asp	55.67	1076	GF-D8	rs121434596	Likely pathogenic	ClinVar
	c.182A>G	p.Gln61Arg	52.63	1520	KMOE-2	rs11554290	Likely pathogenic	ClinVar

	c.181C>A	p.Gln61Lys	33.39	620	M-07e	rs121913254	Likely pathogenic	ClinVar
	c.183A>T	p.Gln61His	45.18	1007	ME-1	rs121913255	Likely pathogenic	ClinVar
	c.35G>A	p.Gly12Asp	51.77	1949	MOLM-20	rs121913237	Likely pathogenic	ClinVar
	c.182A>T	p.Gln61Leu	100.00	468	OCI-AML3	rs11554290	Likely pathogenic	ClinVar
	c.35G>A	p.Gly12Asp	78.57	3145	THP-1	rs121913237	Likely pathogenic	ClinVar
EZH2	c.2215_2219del5	p.Leu739ValfsTer22	49.88	1269	AML-193	NA	Pathogenic	Current data set
	c.1856T>C	p.Leu619Pro	23.82	722	K-562	ND	VUS	Current data set
	c.892C>A	p.Arg298Ser	69.4	794	KG-1a	ND	VUS	Current data set
	c.1851G>T	p.Lys617Asn	99.1	1224	OCI-AML1	ND	VUS	Current data set
	c.2069G>A	p.Arg690His	98.33	5255	OCI-AML5	rs1554481435	Likely pathogenic	ClinVar
KRAS	c.182A>G	p.Gln61Arg	43.96	414	CTS	rs121913240	Pathogenic	ClinVar
	c.35G>T	p.Gly12Val	45.06	2630	MUTZ-2	rs121913529	Pathogenic	ClinVar
	c.29_31dupGAG	p.Gly10dup	29.03	2253	MUTZ-3	COSM12654	VUS	COSMIC
	c.53C>A	p.Ala18Asp	71.95	1843	NB-4	COSM542	VUS	COSMIC
	c.38G>A(v)	p.Gly13Asp	53.85	2951	NOMO-1	rs112445441	Pathogenic	ClinVar
FLT3 exon 14/15	c.1669G>A	p.Val557Ile	99.49	2746	KG-1	rs35958982	Benign	ClinVar
	c.1669G>A	p.Val557Ile	99.5	1210	KG-1a	rs35958982	Benign	ClinVar
	c.1772_1801dup30	p.Leu601_Lys601ins HisValAspPheArgGluTyrGluTyrAsp	76.81	2204	MV4-11	COSM27908	Pathogenic	COSMIC
	c.1775_1795dup21	p.Tyr599_Asp599ins PheAspPheArgGluTyrGlu	53.4	1811	MOLM-14	COSM19953	Pathogenic	COSMIC
	c.1775T>C	p.Val592Ala	67.19	1286	MONO-MAC-1	rs1057520025	Likely pathogenic	ClinVar
FLT3 exon 20	c.2459C>A	p.Thr820Asn	61.65	4621	KMOE-2	COSM2070126	VUS	COSMIC
	c.2459C>A	p.Thr820Asn	49.6	3101	ME-1	rs554971395	VUS	ClinVar
RUNX1	c.419_420delAC	p.Tyr140PhefsTer3	51.67	658	HT-93	NA	Pathogenic	Current data set
	c.401C>T	p.Ala134Val	42.78	1052	MONO-MAC-1	ACH-001129	VUS	CCLE[3]
	c.610C>T	p.Arg204Ter	48.96	1342	MUTZ-2	rs1569061768	Pathogenic	ClinVar
	c.438_442dupTGCTA	p.Thr148MetfsTer6	48.35	4705	OCI-AML5	COSM2844337	Pathogenic	COSMIC
TET2	c.558delG	p.Ser189ValfsTer18	50.73	4481	MOLM-20	NA	Pathogenic	Current data set
	c.3061_3062insA	p.Ser1023GlufsTer4	48.33	1854	MOLM-20	NA	Pathogenic	Current data set
	c.4161C>G	p.Asn1387Lys	16.89	876	MUTZ-3	ND	VUS	Current data set
	c.4541_4542delTT	p.Leu1515AlafsTer62	24.43	839	MUTZ-3	NA	Pathogenic	Current data set
	c.3646C>T	p.Arg1216Ter	49.9	1038	OCI-AML1	COSV54398925	Pathogenic	COSMIC
	c.3009G>A	p.Trp1003Ter	51.26	1746	OCI-AML1	COSV54426154	Pathogenic	COSMIC

	c.2474C>A	p.Ser825Ter	53.36	7953	OCI-AML5	COSM2952770	Pathogenic	COSMIC
WT1	c.1202_1203delGA	p.Arg401IlefsTer3	49.33	673	CTS	NA	Pathogenic	PMID 18591546
	c.1202_1203delGA	p.Arg401IlefsTer3	44.92	2400	KASUMI-6	ACH-000166	Pathogenic	CCLE
	c.1105C>T	p.Arg369Ter	43.11	392	U-937	rs1423753702	Pathogenic	ClinVar
	c.1385G>A	p.Arg462Gln	37.5	2878	MOLM-1	ACH-001573	Likely pathogenic	CCLE
BCOR	c.4988G>A	p.Trp1663Ter	99.42	688	KG-1	ND	Pathogenic	Current data set
	c.871_895del25	p.Met291LeufsTer	95.75	471	MOLM-14	NA	Pathogenic	Current data set
	c.3694C>T	p.Gln1232Ter	99.35	461	MUTZ-2	ND	Pathogenic	Current data set
GATA2	c.344_351delCCTGGACC	p.Pro115ArgfsTer67	33.09	556	M-07e	NA	Pathogenic	CCLE
	c.919C>T	p.Arg307Trp	35.79	2515	MUTZ-3	COSM306052	VUS	COSMIC
BCORL1	c.2350C>T	p.Arg784Ter	65.31	516	AML-193	COSM1319521	Pathogenic	COSMIC
	c.4258C>T	p.Arg1420Ter	98.1	263	MUTZ-2	COSM5880048	Pathogenic	COSMIC
CEBPa	c.917G>C	p.Arg306Pro	48.07	1712	KASUMI-6	ACH-000166	VUS	CCLE
	c.584_589dupACCCGC	p.His195_Pro196dup	23.26	331	THP-1	NA	VUS	Current data set
DNMT3A	c.2462A>G	p.His821Arg	14.23	1286	MUTZ-3	ND	VUS	Current data set
	c.2516C>G	p.Ser839Cys	13.53	4309	MUTZ-3	ND	VUS	Current data set
	c.2644C>T	p.Arg882Cys	60.22	465	OCI-AML3	rs377577594	Pathogenic	ClinVar
NOTCH1	c.7327_7328insGGAAGAGGATG	p.Val2443GlyfsTer38	60.78		AML-193	ACH-000557	Pathogenic	CCLE
	c.6454G>C	p.Gly2152Arg	41.58	1253	OCI-AML5	rs116317506	Benign	ClinVar
NPM1	c.859_860insTCTG	p.Trp288CysfsTer12	12.24	2459	MUTZ-3	rs587776806	Pathogenic	ClinVar
	c.860_863dupTCTG	p.Trp288CysfsTer12	52.89	743	OCI-AML3	rs587776806	Pathogenic	ClinVar
STAG2	c.3308G>A	p.Trp1103Ter	99.44	1440	KASUMI-6	ACH-000166	Pathogenic	CCLE
	c.2009delA	p.Asp671IlefsTer21	96.05	532	MUTZ-2	NA	Pathogenic	Current data set
ATRX	c.5823_5825delTAG	p.Ser1942del	60.37	1986	MOLM-20	NA	VUS	Current data set
CSF3R	c.1853C>T	p.Thr618Ile	43.3	224	MOLM-20	rs796065343	Likely pathogenic	ClinVar
ETV6 /TEL	c.1058G>C	p.Arg353Pro	49.07	2360	AML-193	ACH-000557	VUS	CCLE
JAK2	c.1849G>T	p.Val617Phe	99.62	6287	HEL	rs77375493	Pathogenic	ClinVar
KIT	c.2466T>A	p.Asn822Lys	98.82	1526	SKNO-1	rs121913514	Pathogenic	ClinVar
PHF6	c.618_619insGGGA	p.Asp207GlyfsTer6	59.46	624	AML-193	ACH-000557	Pathogenic	CCLE
PTEN	c.387_388insCGCC	p.Thr131ProfsTer50	96.17	287	U-937	rs121909224	Pathogenic	ClinVar
PTPN11	c.178G>C	p.Gly60Arg	46.54	838	U-937	rs397507507	Uncertain significance	ClinVar
SETBP1	c.2602G>A	p.Asp868Asn	52.8	1053	MOLM-20	rs267607042	Pathogenic	ClinVar
SF3B1	c.1998G>T	p.Lys666Asn	34.78	851	MUTZ-3	rs377023736	Likely pathogenic	ClinVar
U2AF1	c.470A>C	p.Gln157Pro	47.52	322	MONO-MAC-1	rs371246226	Likely pathogenic	ClinVar

Abbreviations. AA, amino acid sequence; VF, variant frequency; ins, insertion; del, deletion; dup, duplication; Ter, termination codon; VUS, variant of unknown significance; CCLE, cancer cell line encyclopedia.

*Transcript IDs used for the analysis: *TP53,* NM_000546.5; *ASXL1,* NM_015338.5*; NRAS,* NM_002524.4*; EZH2,* NM_004456.4*; KRAS,* NM_033360.2*; FLT3,* NM_004119.2*; RUNX1,* NM_001754.4; *TET2,* NM_001127208.2; *WT1,* NM_024426.4; *BCOR,* NM_001123385.1*; GATA2,* NM_032638.4*; BCORL1,* NM_021946.4; *CEBPa,* NM_004364.3; *DNMT3A,* NM_022552.4; *NOTCH1,* NM_017617.3*; NPM1;* NM_002520.6, *STAG2*, NM_001042749.1; *ATRX,* NM_000489.3; *CSF3R*, NM_156039.3; *ETV6/TEL*, NM_001987.4; *JAK2,* NM_004972.3; *KIT,* NM_000222.2*; PHF6,* NM_032458.2; *PTEN,* NM_000314.4; *PTPN11,* NM_002834.3; *SETBP1,* NM_015559.2; *SF3B1,* NM_012433.2; *U2AF1,* NM_001025203.1.

[1]ClinVar database accessed at https://www.ncbi.nlm.nih.gov/clinvar; [2]COSMIC database accessed at https://cancer.sanger.ac.uk/cosmic; 2CCLE database accessed at https://depmap.org.

Table S6. Overview of patient characteristics and genomic background.

P#	Gender	Age	Blast (%)[1]	Diagnosis	Karyotype	Co-occurring Genetic Mutations			
						FLT3-ITD	*KIT*	*NPM1*	*nRAS*
1	m	14	88.5	AML M1	46,XY,t(1;2)(p35;p22)[14]/46,XY[2]	Yes	No	No	No
2	f	5	91	AML M4	46,XX,add(2)(q?34)[14]/46,XX[6]	No	No	No	No
3	f	11	95	AML M1	46,XX[25]	No	No	No	No
4	f	12	41	AML M4	46,XX[25]	No	No	No	No
5	f	3	55	AML M1	46,XX[15]	No	No	No	No
6	f	9	91	AML	46,XX,t(5;11)(q35;p15)[20]	Yes	No	No	No
7	m	8	93	AML M1	46,XY,der(14)t(14;17)(p11;q21)[7]/46,idem,del(3)(q13q26)[1]/47,idem,del(3)(q13q26),+4[1]/46,XY[9]	No	No	No	No
8	f	12	90	AML M1/M2	46,XX[25]	Yes	No	No	No
9	m	13	95	AML M1	46,XY,del(9)(q11q?33)[17]/46,XY[3]	No	No	No	No
10	m	5	51	AML M5	46,XY[25]	Yes	No	No	No
11	m	8	78	AML M2/M4	46,XY,add(11)(q23),inc	No	No	No	No
12	f	1	37	AML M4	46,XX,inv(16)(p13q22)	No	No	No	Yes
13	m	17	76	AML M2	46,XY[12]	Yes	No	No	No
14	m	17	35	AML M5	46,XY[25]	Yes	No	Yes	No
15	f	16	79	AML M4	45,XX,inv(3)(q21q26),-7[25]/46,XX[1]	No	No	No	No
16	m	14	84	AML M0	46,XY,add(2)(pl4),add(4)(q,26),der(8)t(8;12)(p12;q12),-12,add(16)(q21),+17[15]/46,XY[5]	No	Yes	No	No
17	m	16	67	AML M1	45,XY,-7[20]	No	No	Yes	Yes
18	m	14	86	AML M4	46,XY[15]	Yes	No	Yes	No
19	m	14	66	AML M4eo	46,XY[25]	Yes	No	No	No
20	m	5	33	AML M2	46,XY[20]	No	No	No	No
21	m	9	57	AML M4	47,XY,+8,t(16;16)(p13;q22)[13]/48,idem,+22[2]	No	Yes	No	Yes
22	m	9	62	AML M1/M2	47,XY,+8[7]/46,XY[11]	Yes	No	No	No
23	f	16	90	AML M0	54,XX,+4,+8,+13,+18,+19,+20,+21,+22[16]/46,XX[4]	No	No	No	No
24	m	6	88	AML M1/M2	46,XY[25]	Yes	No	No	No

Abbreviations. P#, patient number; m, male; f, female. [1]Percentage of bone marrow malignant blasts at the time of diagnosis.
